# Morphological and Phylogenetic Analyses Reveal Five New Species in Chaetosphaeriaceae

**DOI:** 10.3390/jof8060643

**Published:** 2022-06-17

**Authors:** Jing-Yi Zhang, Jian Ma, Yuan-Pin Xiao, Saranyaphat Boonmee, Ji-Chuan Kang, Yong-Zhong Lu

**Affiliations:** 1 School of Food and Pharmaceutical Engineering, Guizhou Institute of Technology, Guiyang 550003, China; zjingyi127@gmail.com (J.-Y.Z.); yanmajian@163.com (J.M.); emmaypx@gmail.com (Y.-P.X.); 2 Center of Excellence in Fungal Research, Mae Fah Luang University, Chiang Rai 57100, Thailand; saranyaphat.boo@mfu.ac.th; 3 School of Science, Mae Fah Luang University, Chiang Rai 57100, Thailand; 4 Guizhou Key Laboratory of Agricultural Biotechnology, Guizhou Academy of Agricultural Sciences, Guiyang 550006, China; 5 Engineering and Research Center for Southwest Bio-Pharmaceutical Resources of National Education Ministry of China, Guizhou University, Guiyang 550025, China; jckang@gzu.edu.cn

**Keywords:** 8 taxa, asexual morph, Chaetosphaeriales, hyaline spore, taxonomy

## Abstract

Chaetosphaeriaceae is a genera-rich and highly diverse group of fungi with a worldwide distribution in terrestrial and aquatic habitats. Eight fresh collections of Chaetosphaeriaceae were obtained during investigations of hyaline-spored hyphomycetes in China and Thailand. Based on morphological characteristics and phylogenetic analysis of a combined LSU and ITS sequence dataset, *Chaetosphaeria obovoidea*, *Codinaea aseptata*, *Codinaeella hyalina*, *Dictyochaeta guizhouensis* and *Paragaeumannomyces guttulatus* were introduced as new species, *Codinaea terminalis* was reported as new host record, and *Codinaea dwaya* and *Phialosporostilbe scutiformis* were introduced as new collections. Phylogenetic analysis in this study revealed that *Chaetosphaeria* was polyphyletic. Detailed descriptions and illustrations of new taxa and identified species are provided, as well as an updated phylogenetic tree to confirm the placements of these eight new collections.

## 1. Introduction

Reblova et al. [1] validly established the Chaetosphaeriaceae to accommodate type species *Chaetosphaeria* Tul. and C. Tul. and their relatives, including *Ascocodinaea*, *Melanochaeta*, *Melanopsammella*, *Porosphaerella*, *Porosphaerellopsis* and *Striatosphaeria*. Subsequently, more new taxa were added to this family [2,3,4,5,6,7,8,9,10]. Reviews of Chaetosphaeriaceae were provided by Lin et al. [9] and Hyde et al. [11]. Forty-three genera were accepted in Chaetosphaeriaceae by Hyde et al. [11]. In recent studies, Zheng et al. [12] included a new genus *Phialolunulospora* in the family. Réblová et al. [13,14] introduced six new genera namely *Arcuatospora*, *Achrochaeta*, *Ericiosphaeria*, *Flectospora*, *Phialoturbella* and *Tubulicolla*. The lineages of “*Catenularia*”-like taxa were recognized as a genus *Fuscocatenula* by Réblová et al. [15]. Five lineages, *Codinaeella*, *Nimesporella*, *Stilbochaeta*, *Tainosphaeriella* and *Xyladelphia*, were proposed for “*Codinaea*”-like fungi by Réblová et al. [16]. Meanwhile, the key to asexual genera of Chaetosphaeriaceae was provided by Maharachchikumbura et al. [17].

*Chaetosphaeria* (Chaetosphaeriaceae, Chaetosphaeriales), was established by Tulasne and Tulasne [18] with the type species *C*. *innumera* Berk. and Broome ex Tul. and C. Tul. It has simple and homogeneous sexual morphs, and complex and diverse asexual morphs [3,4,5,9,10,11,19,20]. Its members have been shown to link with several asexual genera in Chaetosphaeriaceae, including *Chloridium*, *Codinaea*, *Dictyochaeta* and *Menispora* [9,21,22]. Following the “One Fungus One Name” (1F1N) principle, some *Chaetosphaeria* species were synonymized under respective anamorphic genera, the latter being older and prioritized, i.e., *Cacumisporium*, *Catenularia*, *Chloridium*, *Codinaea*, *Dictyochaeta*, *Exserohilum*, *Gonytrichum* and *Menispor* [4,13,14,15,23,24].

*Codinaea* was established by Maire [25] and typified by *C*. *aristata* Maire. Gamundi et al. (1977) synonymized *Codinaea* under *Dictyochaeta* Speg. based on a misidentification of *D*. *fuegiana* Speg., the type species of *Dictyochaeta*. Based on the morphological reexamination of *D*. *fuegiana*, Réblová [21] argued that *Codinaea* and *Dictyochaeta* were separate genera in Chaetosphaeriaceae. She proposed that *Codinaea* species have conidia with setulae, while members of *Dictyochaeta* do not. This proposal was accepted in later studies [11,14,26,27,28,29].

*Codinaeella* is established by Réblová et al. [16] for “*Codinaea*”-like fungi based on molecular data and morphological characters of setae and conidiophores, which is typified by *Cod*. *minuta* (Tubaki) Réblová and Hern.-Restr. *Codinaeella* species have a saprobic life mode on various plants and soil [16,30,31]. Réblová et al. [16] accepted eight species in *Codinaeella* based on molecular data and morphological evidence, and provided a synopsis of accepted species in *Codinaeella* [16,30,32].

*Dictyochaeta* was introduced by Spegazzini [32] and typified by *D*. *fuegian*. The genus has been considered polyphyletic and phylogenetically unresolved due to its long-term broad delimitation [3,8,9,29,33]. Later, Réblová et al. [14] indicated that *Dictyochaeta* sensu stricto including *D. callimorpha*, *D. detriticola*, *D*. *fuegiana* (type species), *D. montana, D. querna* and *D. stratosa* formed a monophyletic clade in Chaetosphaeriaceae based on combined ITS and LSU sequence analysis. Furthermore, Réblová et al. [14] reevaluated a generic concept of *Dictyochaeta* based on multi-loci analyses coupled with morphological comparison and culture characteristics. The keys to the accepted *Dictyochaeta* species were provided by Kuthubutheen and Nawawi [33] and Réblová et al. [14].

*Paragaeumannomyces* was typified by *P*. *sphaerocellularis* Matsush [34]. *Paragaeumannomyces* in Chaetosphaeriaceae was confirmed as a monophyletic clade based on the phylogenetic analyses of combined ITS and LSU sequence data [29]. The key to the 16 accepted *Paragaeumannomyces* species was provided by Réblová et al. [29]. Furthermore, Réblová et al. [13] introduced the new combination *Paragaeumannomyces hispidus* (Réblová and Seifert) Réblová and Hern.-Restr. (basionym: *Chaetosphaeria hispida* Réblová and Seifert) based on characteristics of a three-layered ascomatal wall. It is difficult to distinguish *Paragaeumannomyces* from the similar genus *Ericiosphaeria* morphologically, as they share semblable morphology in asci, ascospores and setae. However, the two genera can be distinguished based on a number of layers and texture of the ascomatal wall as well as molecular data [6,13,29].

The hyphomycetous genus *Phialosporostilbe* was verified by Sierra and Portales [35] to accommodate *P*. *turbinate* Mercado and J. Mena. The placement of *Phialosporostilbe* in Chaetosphaeriaceae was confirmed by Yang et al. [36], based on morphological and phylogenetic analyses of the combined ITS and LSU sequence data. The genus is characterized by synnematous conidiophores, monophialidic conidiogenous cells and hyaline, turbinate or cordiform conidia with subapical or apical appendages [36,37,38]. *Phialosporostilbe* can be distinguished from the similar genus *Nawawia* by its synnematous conidiophores [35,36,37]. Index Fungorum (May 2022) listed seven epithets under *Phialosporostilbe*, of which only one species has molecular data available.

In this study, eight chaetosphaeriaceous hyphomycetes were obtained from freshwater and terrestrial habitats in China and Thailand, and eventually identified five novel species (*Chaetosphaeria obovoidea*, *Codinaea aseptata*, *Codinaeella hyalina*, *Dictyochaeta guizhouensis* and *Paragaeumannomyces guttulatus*), a new host record *Codinaea terminalis* and two new collections of *Codinaea dwaya* and *Phialosporostilbe scutiformis*. These new isolated data are provided with details (Table 1). Morphological evidence and phylogenetic analysis based on a combined LSU and ITS sequence dataset confirmed the identity of these new collections, providing further evidence on the phylogenetic placement of these taxa in Chaetosphaeriaceae.

## 2. Materials and Methods

### 2.1. Collection, Isolation and Conservation

Samples of submerged decaying twigs and dead bamboo culms were collected from Chiang Rai Province, Thailand, and Guizhou Province, China. They were packed into plastic bags for transportation to the laboratory, and associated metadata (i.e., date, locality and host) were noted. Fungal colonies on the host surface were examined and observed using a stereomicroscope (Leica EZ4 Microsystems (Schweiz) AG, Singapore). Photomicrographs of micro-morphological characteristics were documented using a Nikon DS-Ri2 digital camera fitted to a Nikon ECLIPSE Ni compound microscope (Nikon, Japan). Measurements were made using the Tarosoft (R) Image Frame Work, and these images used for figures were processed and combined with Adobe Illustrator CS6 (Adobe Systems, San Jose, CA, USA).

Single-spore isolations were made on water agar (WA) and potato dextrose agar (PDA), and germinating conidia were transferred to fresh malt extract agar (MEA) and PDA following the method of Chomnunti et al. [39]. Dried materials were deposited in the herbaria of Mae Fah Luang University (Herb. MFLU), Chiang Rai, Thailand, Herbarium of Cryptogams, Kunming Institute of Botany, Academia Sinica (HKAS), Kunming, China, and Herbarium of Guizhou Academy of Agricultural Sciences (GZAAS), Guiyang, China. Cultures were deposited at Mae Fah Luang University Culture Collection (MFLUCC), Chiang Rai, Thailand, and Guizhou Culture Collection, China (GZCC). Faces of Fungi and Index Fungorum numbers were registered according to the guidelines in Jayasiri et al. [40] and Index Fungorum (2022).

### 2.2. DNA Extraction, PCR Amplification and Sequencing

Pure cultures were grown on MEA/PDA media at 25 °C for one month. Fresh fungal mycelia were scraped off from the surface of the cultures and transferred to 1.5 mL microcentrifuge tubes. Meanwhile, fungal genomic DNA was extracted with the Biospin Fungus Genomic DNA Extraction Kit (Biospin Fungus Genomic DNA Extraction Kit, BioFlux^®^, Shanghai, China) following the manufacturer’s instructions. LR0R and LR5 (Vilgalys and Hester 1990) and ITS5 and ITS4 (White et al. 1990) primers were used to amplify the large subunit of the ribosomal DNA (LSU) and the internal transcribed spacer (ITS) gene regions. The amplification reactions were performed in a 50 μL reaction volume, which contained 2 μL of DNA template, 2 μL of each forward and reverse primer (10 μM), 25 μL of 2× Taq PCR Master Mix with blue dye (Sangon Biotech, Shanghai, China) and 19 μL of distilled–deionized water. The following thermo-cycling parameters were used for the LSU and ITS region: initial denaturation at 94 °C for 3 min, followed by 35 cycles of denaturation at 94 °C for 45 s, annealing at 56 °C for 50 s, elongation at 72 °C for 1 min and a final extension period for 10 min at 72 °C. The quality of the PCR products was checked on a 1% agarose gel electrophoresis stained with ethidium bromide. Purification and sequencing of PCR products were performed at Sangon Biotech (Shanghai, China) using the same primers.

### 2.3. Alignments and Phylogenetic Analysis

Original sequences were verified using BioEdit v. 7.1.3.0 [41], and were assembled using SeqMan v. 7.0.0 (DNASTAR, Madison, WI, USA). Consensus sequences were submitted to the NCBI GenBank (Table 1). The new sequences were subjected to BLASTn (https://blast.ncbi.nlm.nih.gov/Blast.cgi, accessed on 1 May 2022) for preliminary determination of the possible species identification range in the GenBank database. Sequences of the ITS and LSU gene were analyzed to assess relationships among species of *Chaetosphaeria*, *Codinaea*, *Codinaeella*, *Dictyochaeta*, *Paragaeumannomyces*, *Phialosporostilbe* and relevant fungi in Chaetosphaeriaceae [6,9,10,12,14,16,20,29,36,42]. Accession numbers for sequences were retrieved from GenBank database and related publication from previous studies (Table 2 and Table S1). The alignments for sequences of each locus were performed with the online multiple alignment program MAFFT version 7 (https://mafft.cbrc.jp/alignment/server/, accessed on 3 May 2022) and then manually verified in BioEdit 7.1.3.0 [43]. The maximum likelihood (ML) and Bayesian inference (BI) analyses inferred phylogenetic relationships, based on the concatenated sequence matrix of a combined LSU and ITS dataset.

The website tool “ALTER” (http://www.singgroup. org/ALTER/, accessed on 3 May 2022) was used to convert the aligned fasta file for RAxML analysis [44]. Subsequently, ML analysis was performed using RAxML-HPC v.8 tool via the CIPRES Science Gateway V3.3 (https://www.phylo.org/portal2/home.action, accessed on 3 May 2022) with rapid bootstrap analysis [45,46]. Eventually, 1000 non-parametric bootstrap iterations were run with the GTRGAMMA model, and the final tree was selected amongst suboptimal trees from each run by comparing likelihood scores under the GTR-Gamma substitution model.

The aligned fasta file was converted to the nexus file format for BI analysis using AliView. BI analysis was performed using MrBayes 3.2.7a via CIPRES [45]. The best-fit model of DNA evolution was estimated using MrModeltest v. 2.2 [47]. The Bayesian Markov chain Monte Carlo (BMCMC) sampling method in MrBayes v.3.2.7a determined the posterior probabilities (PPs) [48]. Four simultaneous Markov chains were run for 1 million generations, with trees sampled every 100 generations, resulting in 10,000 trees. The first 2000 trees representing the burn-in phase of the analyses were discarded, and the remaining 8000 trees were used for calculating posterior probabilities (PPs) in the majority-rule consensus tree [49].

Phylogenetic trees were printed with FigTree v. 1.4.0 [50], and the layout was established using Adobe Illustrator CS6 (Adobe Systems, San Jose, CA, USA).

## 3. Results

### 3.1. Phylogenetic Analysis

**Figure 1:**
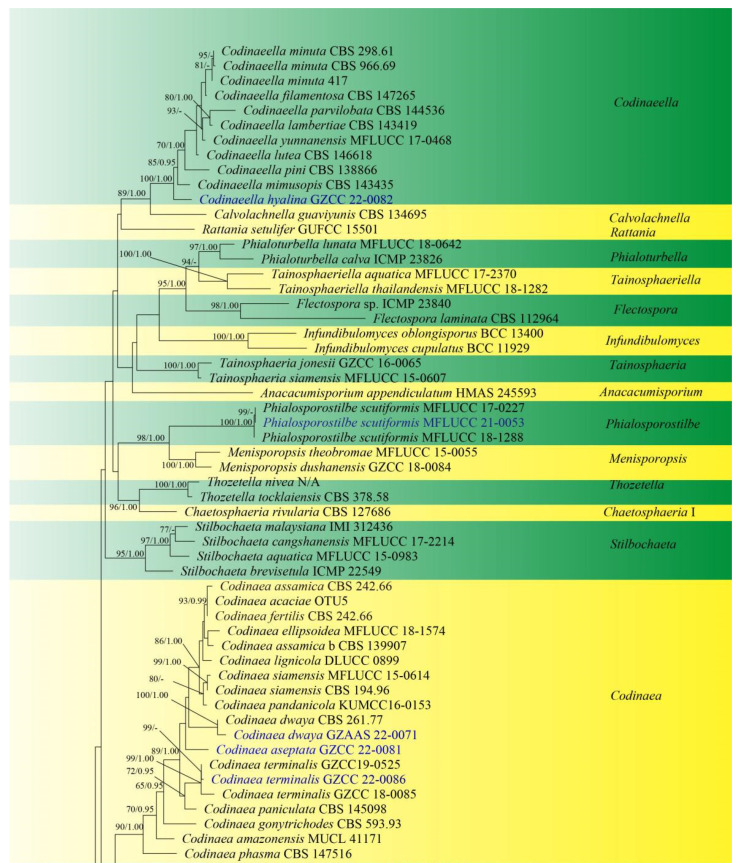

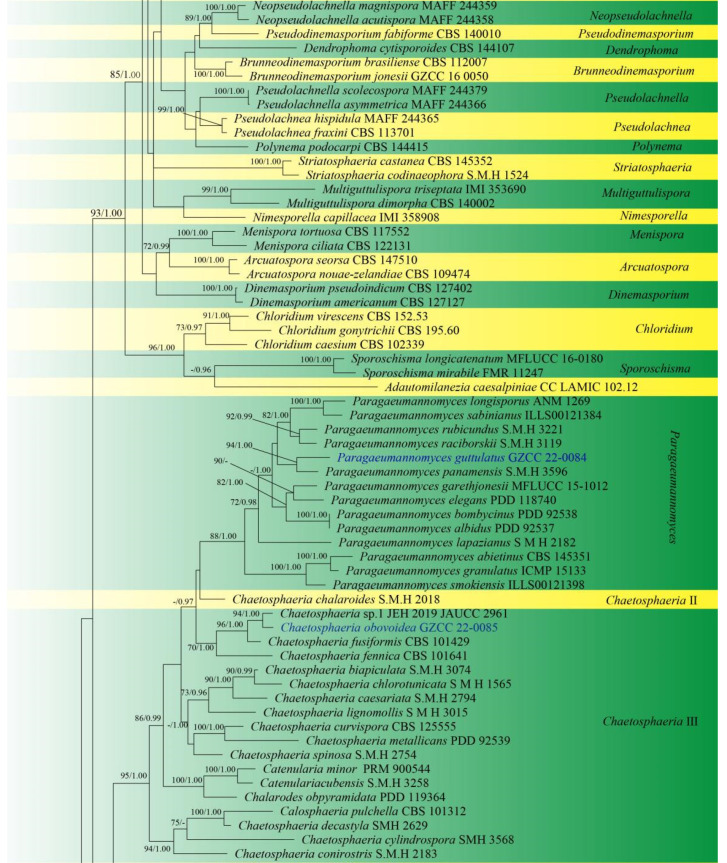

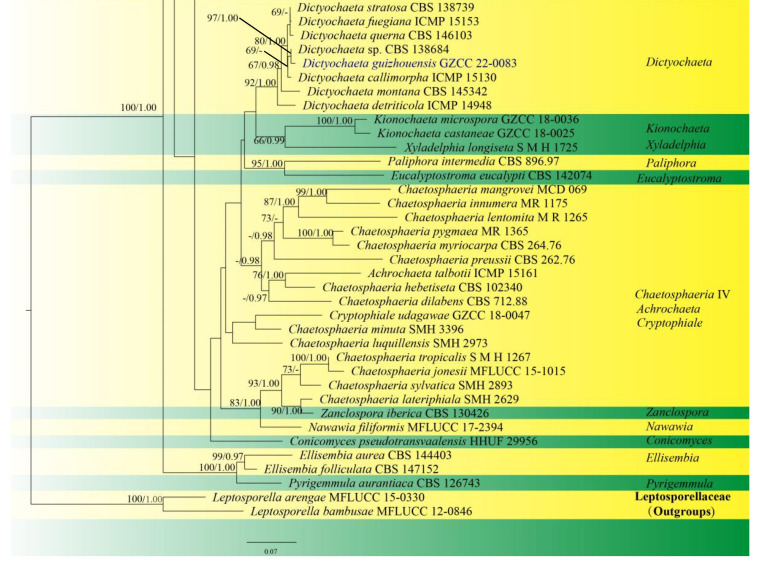
The phylogenetic tree generated from ML analysis that is based on a concatenated LSU-ITS dataset for the Chaetosphaeriaceae family. Bootstrap support values for ML equal to or greater than 65% and Bayesian posterior probabilities (PPs) equal to or greater than 0.95 were indicated above or below the nodes as ML/PP. *Leptosporella arengae* (MFLUCC 15-0330) and *L*. *bambusae* (MFLUCC 12-0846) were selected as the outgroup taxa. The newly obtained sequences are indicated in blue.

Sequences of LSU and ITS were used to determine the phylogenetic position of the new taxa and collections. The concatenated sequence matrix comprises LSU (903 bp) and ITS (545 bp) sequence data for 151 ingroup taxa of Chaetosphaeriaceae and 2 outgroup taxa, namely *Leptosporella arengae* (MFLUCC 15-0330) and *L*. *bambusae* (MFLUCC 12-0846). The sequence matrix comprises 1448 characters after alignment, including the gap. The matrix had 859 distinct alignment patterns, with 14.02% undetermined characters or gaps. The estimated base frequencies were: A = 0.228939, C = 0.268473, G = 0.306331 and T = 0.196257; substitution rates AC = 1.246064, AG = 1.891246, AT = 1.625669, CG = 0.550487, CT = 6.118919, GT = 1.000000 and Tree-Length = 11.191081; distribution shape parameter α = 0.304587. Bayesian posterior probabilities (PPs) from MCMC were evaluated with a final average standard deviation of split frequencies of 0.009954. Maximum likelihood and Bayesian analyses were conducted, resulting in generally congruent topologies, and the ML analysis result with a final likelihood value of −27,871.044543 is presented in Figure 1.

This phylogenetic study confirmed that the family Chaetosphaeriaceae was a robust clade (100% ML/1.00 PP). There are eight new collections, five of which are in *Chaetosphaeria*, *Codinaeella*, *Dictyochaeta*, *Paragaeumannomyces* and *Phialosporostilbe* genera, respectively, while the other three belong to the genus *Codinaea*, under Chaetosphaeriaceae (Figure 1).

*Chaetosphaeria* was resolved as a polyphyletic genus in Chaetosphaeriaceae (Figure 1), consistent with many previous studies [6,9,10,13,14,15,29,51,52]. The new species, *Chaetosphaeria obovoidea*, and three other *Chaetosphaeria* species are clustered in the subclade of *Chaetosphaeria* III.

The phylogenetic tree shows that our three new *Codinaea* collections of *Codinaea aseptata* sp. nov., and two known species, *Co*. *dwaya* and *Co*. *terminalis*, clustered with other 16 *Codinaea* taxa in a monophyletic clade with highly support (90% ML/1.00 PP, Figure 1). 

Eight “*Codinaea*”-like taxa comprised a new species of *Codinaeella hyalina* and eight previously identified species, representing the *Codinaeella* clade, which was phylogenetically well-supported by the generic placement (100% ML/1.00 PP, Figure 1). It is consistent with the studies of Réblová et al. [16].

One of the eight fresh collections was identified as a new species in *Dictyochaeta* species, namely *D*. *guizhouensis*. It clustered together with the other six known species and one unidentified *Dictyochaeta* sp. (CBS 138684) with good support (97% ML/1.00 PP, Figure 1). All species of *Dictyochaeta* formed into a single monophyletic clade and fit well with the narrow genetic concept of conidia without setulae [14]. Furthermore, the phylogenetic placement of the *Dictyochaeta* clade and the phylogenetic relationship of taxa within *Dictyochaeta* presented similar results to those obtained by Réblová et al. [14].

Phylogenetic placements of the *Paragaeumannomyces* and *Phialosporostilbe* genera were stable, and those genera were resolved as monophyletic lineages [29,36].

### 3.2. Taxonomy

**Figure 2:**
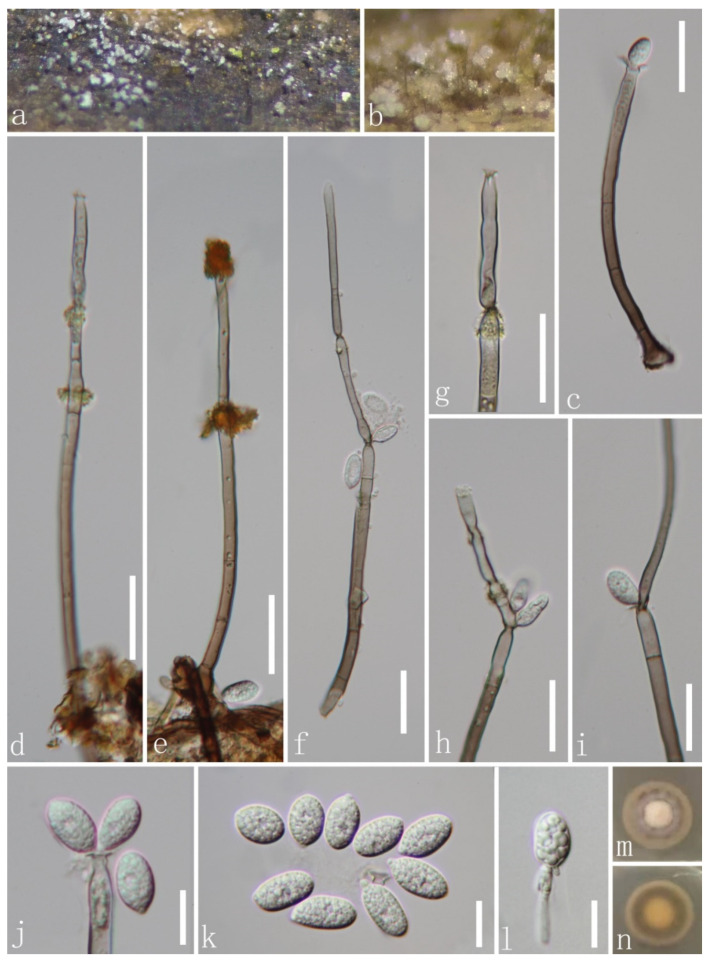
*Chaetosphaeria obovoidea* (HKAS 123765, holotype). (**a**,**b**) Colonies on woody substrate; (**c**–**f**) conidiophores with pale gold or red appendant attached; (**g**–**j**) conidiogenous cells; (**j**–**l**) conidia; (**l**) germinating conidium; (**m**,**n**) culture on PDA from above and below. Scale bars: (**c**–**f**) 25 μm; (**g**–**i**) 20 μm; (**j**–**l**) 10 μm.

*Chaetosphaeria obovoidea* J.Y. Zhang and Y.Z. Lu, sp. nov. (Figure 2).

Index Fungorum number: IF559696; Facesoffungi number: FoF 11036.

Etymology: referring to obovoid conidia.

Holotype: HKAS 123765.

*Saprobic* on decaying wood submerged in freshwater habitats. Sexual morph: undetermined. Asexual morph: hyphomycetous. *Colonies* on natural substrate superficial, effuse, scattered, pale brown, with white and gold masses on the apex of conidiophores. *Mycelium* is composed of partly immersed, partly superficial, hyaline-to-pale brown, septate, branched hyphae. *Conidiophores* 93–234(–291) μm long, 3.5–5.3 μm wide at the base, macronematous, mononematous, erect, straight or slightly flexuous, occasionally branched, with intercalary conidiogenous loci, cylindrical, solitary, brown at the base, pale brown or subhyaline towards the apex, septate, smooth-walled, with pale gold or red appendants attached. *Conidiogenous cells* 13–51 × 3–5.5 μm ($\bar{x}$ = 33.8 × 4.6 μm, *n* = 20), mono- to polyphialidic, terminal, integrated, cylindrical, with funnel-shaped collarettes, brown or pale brown to subhyaline or hyaline towards the apex. *Conidia* 10–14.8 × 5–7.2 μm ($\bar{x}$ = 12.7 × 6 μm, *n* = 20), amerospores, aseptate, obovoid, pyriform to broadly clavate, rough-walled, aggregated in large and slimy mass, hyaline, round at the apex, tapering at the base and often with a small prominence.

Culture characteristics: Conidia germinating on PDA within 15 h and hyaline germ tube produced from the base of conidia. Colonies growing on PDA at 25 °C reach 17 mm in three weeks, circular, unbonate, entire, with filamentous, dense, aerial mycelium on the surface, white at the center, pale grey at the edge from above; yellowish to greyish brown to pale brown in reverse from the center to the margin of the colony, and do not produce pigmentation in culture.

Material examined: CHINA, Hainan province, Diaoluo Mountain National Nature Reserve, on decaying wood submerged in a stream, 20 August 2021, W.G. Lin, DL2 (HKAS 123765, holotype; GZAAS 22-0076, isotype), ex-type living cultures, GZCC 22-0085, GenBank accession numbers: (LSU) ON502894, (ITS) ON502901.

Notes: In a BLASTn search in GenBank, the closest match to the ITS sequence of our new isolate was *Chaetosphaeria* sp. (strain JEH-2019) with 97% (MN619651) similarity across 88% of the query sequence. The closest match to the LSU of the new isolate was *Chaetosphaeria fusiformis* (strain CBS 101429) with 99.21% (AF178554) similarity across 94% of the query sequence. The phylogenetic analysis confirmed that our new isolate of *C*. *obovoidea* formed a separate clade within the *Chaetosphaeria* genus and is a sister clade to *Chaetosphaeria* sp. (strain JEH-2019) with good support (94% ML/1.00 PP). Thus, we introduced this new isolate as a novel species in the *Chaetosphaeria*. Additionally, our new species formed a unique asexual morph in the natural substrate, and differs from all existing species of *Chaetosphaeria* in having macronematous conidiophores with pale gold or red appendants attached, hyaline, aseptate and obovoid conidia with a prominence at the base. This adds complexity and diversity to the asexual morphs of the *Chaetosphaeria* genus.

**Figure 3:**
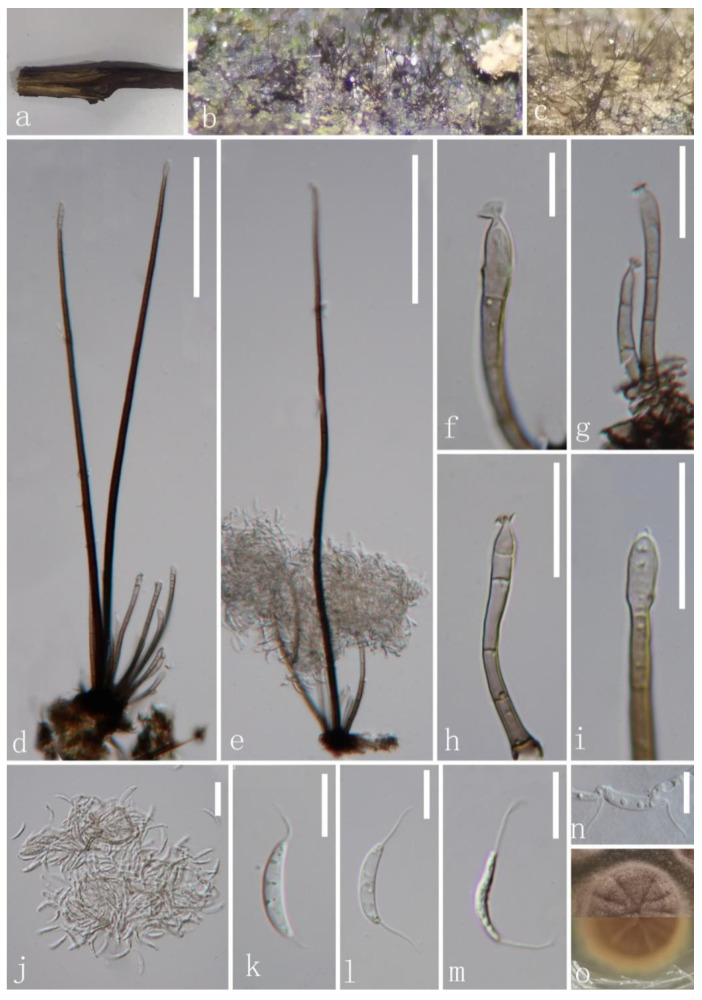
*Codinaea aseptata* (HKAS 123758, holotype). (**a**) Host material; (**b**,**c**) colonies on woody substrate; (**d**,**e**) setae and conidiophores; (**f**–**h**) conidiophores and conidiogenous cells; (**i**) apex of seta;(**j**–**m**) conidia; (**n**) germinated conidium; (**o**) culture on PDA from above and below. Scale bars: (**d**,**e**) 100 μm; (**f**,**k**–**n**) 10 μm; (**g**–**j**) 20 μm.

*Codinaea aseptata* J.Y. Zhang and Y.Z. Lu, sp. nov. (Figure 3).

Index Fungorum number: IF559697; Facesoffungi number: FoF 11038.

Etymology: referring to the aseptate conidia.

Holotype: HKAS 123758.

*Saprobic* on decaying wood submerged in freshwater habitats. Sexual morph: undetermined. Asexual morph: hyphomycetous. Colonies on the natural substrate effuse, hairy, brown. *Mycelium* mostly immersed, composed of branched, septate, smooth, brown hyphae. *Setae* 339–417 μm long, 5–6 μm wide at the base ($\bar{x}$ = 376.8 × 5.5 μm, *n* = 20), erect, dark brown at the base, subhyaline towards the apex, septate, unbranched, smooth, rarely fertilizable. *Conidiophores* 41.6–105.1 μm long, 2–3.74 μm wide at the base ($\bar{x}$ = 66 × 3 μm, *n* = 15), macronematous, mononematous, in groups from the mycelial knots associated with the bases of setae, shorter than setae, erect, straight or slightly flexuous, short, cylindrical, unbranched, smooth-walled, septate, pale brown at the base, subhyaline at the apex. *Conidiogenous cells* 5.7–20 μm ($\bar{x}$ = 12.2 μm, *n* = 20) long, monophialidic, integrated, determinate, terminal, cylindrical to cylindrical–lageniform, pale brown at the base, becoming subhyaline to hyaline towards the apex, with flared collarette. *Conidia* 13.4–16.2 × 2.1–3 μm ($\bar{x}$ = 14.2 × 2.6 μm, *n* = 20), acrogenous, solitary, aggregated in slimy droplets, aseptate, cylindrical or long fusiform, curved, with hair-like and 7–10 m-long appendages at both ends, hyaline, smooth-walled, with small guttules.

Culture characteristics: Conidia germinating on PDA within 15 h and germ tubes produced from the base and the upper part. Colonies growing on PDA, reaching 19 mm in diam. in 10 days at 25 °C, circular, flat, entirely to slightly filamentous, section to fan shape at the surface, taupe, becoming white towards the edge from above; yellowish-brown mycelium in the middle and pale-yellow mycelium in the outer ring in reverse, and does not produce pigmentation in culture. 

Material examined: CHINA, Hainan Province, Wuzhishan City, Wuzhishan Tropical Rainforest Scenic Area, on decaying wood submerged in a freshwater stream, 15 August 2021, Zheng Luo, WZ48 (HKAS 123758, holotype; GZAAS 22-0072 isotype); ex-type living cultures, GZCC 22-0081. GenBank accession numbers: (LSU) ON502890, (ITS) ON502897.

Notes: Four morphotypes (C1–C4) of *Codinaea* were provided by Réblová et al. [16]. The morphological characteristics of our new isolate match well with the generic concept of *Codinaea* and fit well with the morphotype C1. *Codinaea aseptata* is most similar to *Co*. *terminalis* due to having setae in fascicles with conidiophore, phialidic and terminal conidiogenous cells and aseptate, falcate conidia with setulae at both ends [9]. However, our new isolate differs from *Co*. *terminalis* in its obviously longer and rarely fertilizable setae. BLAST results of ITS and LSU sequence data are *Codinaea* *acacia* OTU5 (96.09% similarity) and *Co*. *paniculate* MFLU 34876 (99.43 similarity), respectively. The phylogenetic tree (Figure 1) showed that our new isolate of *Cod*. *aseptata* formed an individual lineage in the *Codinaea* clade, but without statistical support shown in phylogenetic tree (44% ML/-). This may be due to the consideration that many close phylogenetic relatives of our new collection have not yet been discovered. Hence, *Codinaea aseptata* was introduced as a new species based on its distinct morphology and phylogenetic evidence.

**Figure 4:**
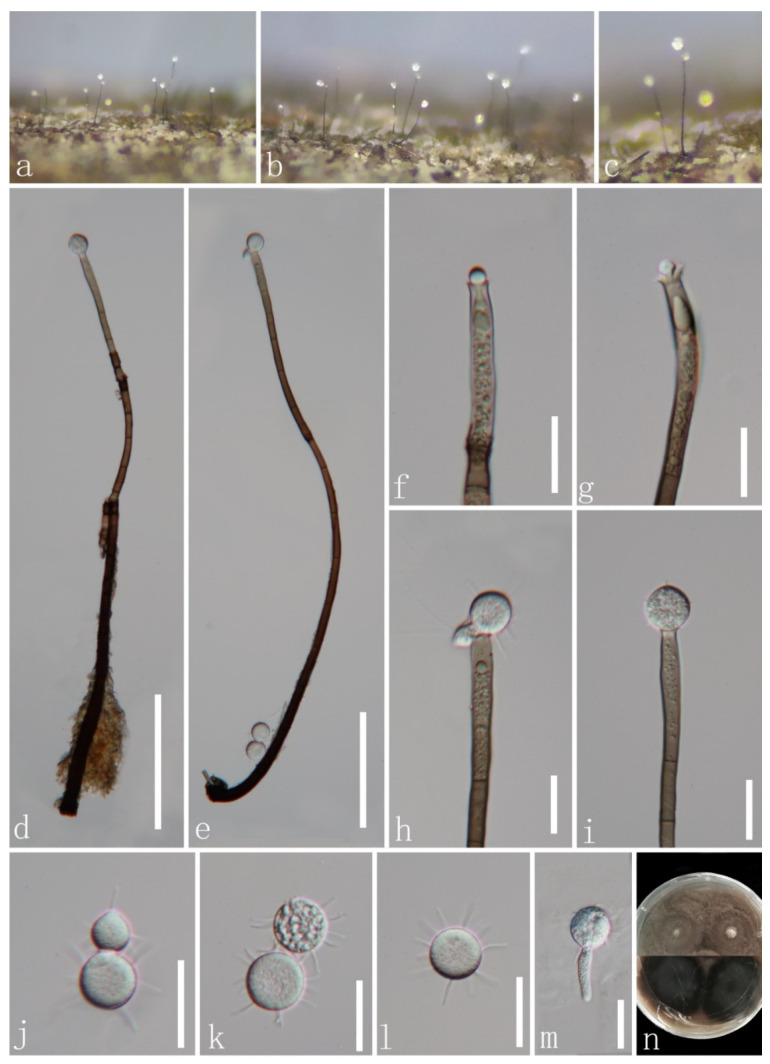
*Codinaea dwaya* (GZAAS 22-0071). (**a**–**c**) Colonies on woody substrate; (**d**,**e**) conidiophores and conidia; (**f**–**i**) conidiogenous cells; (**j**–**l**) conidia; (**m**) germinating conidium; (**n**) culture on PDA. Scale bars: (**d**,**e**) 100 μm; (**f**–**m**) 20 μm.

*Codinaea dwaya* (Subram. and J. Bhat) Réblová and Hern.-Restr., Journal of Fungi 7 (12, no. 1097): 31 (2021) (Figure 4).

Index Fungorum number: IF 842192; Facesoffungi number: FoF 11037.

Holotype: MUBL 2351.

*Saprobic* on decaying wood submerged in freshwater habitats. Sexual morph: undetermined. Asexual morph: hyphomycetous. *Colonies* on natural substrate superficial, effuse, scattered, brown, with glistening masses of conidia on the apex of conidiophores. *Mycelium* 1.3–2.4 μm wide, mostly immersed, composed of pale brown, septate hyphae. *Conidiophores* (360–)440–620 × 7.6–14 μm ($\bar{x}$ = 527.7 × 9.9 μm, *n* = 20), macronematous, mononematous, erect, straight or slightly flexuous, occasionally branched, cylindrical, solitary, septate, smooth-walled, dark brown, becoming paler brown towards the apex. *Conidiogenous cells* (17–)35–62.5 × 5.6–8 μm ($\bar{x}$ = 50.2 × 6.6 μm, *n* = 20), monophialidic, terminal, integrated, cylindrical, guttulata, with an inconspicuous apical collarette. *Conidia* in succession by percurrent proliferation from a single fertile locus, spherical, 13.4–17 μm diam. ($\bar{x}$ = 15.5 μm), amerospores, aseptate, acrogenous, in groups, accumulating in a slimy mass at the tip of the phialide, rough-walled, hyaline, with 7–13 hair-like and 6.4–9.9 μm-long appendages at both ends.

Culture characteristics: Conidia germinating on PDA within 16 h and germ tube produced from conidia. Colonies growing on PDA, reaching 25 mm in diam. in 10 days at 25 °C, circular. Mycelium slightly raised, taupe dark brown with a white protuberance in the center, entirely to slightly filamentous, becoming taupe to dark brown towards the edge in reverse and not producing pigmentation in culture.

Material examined: CHINA, Hainan province, Diaoluo Mountain National Nature Reserve, on decaying wood submerged in a freshwater stream, 20 August 2021, Wei-Guo Lin, DL1-1 (GZAAS 22-0071), living cultures, GZCC 22-0080, GenBank accession numbers: (LSU) ON502889, (ITS) ON502896.

Notes: Following BLASTn searches of NCBI GenBank, the closest matches of the LSU and ITS sequences of our new isolate is *Codinaea dwaya* (strain CBS 261.77; LSU, OL654135, 99.88% shared identity; ITS, OL654078, 97.79% shared identity). Our new collection fits well with the description of species of *Co*. *dwaya* in mononematous conidiophores, integrated, terminal, cylindrical, phialidic conidiogenous cells and hyaline, spherical, aseptate conidia with setulae [16,53,54,55]. Phylogenetically, our isolate grouped together with *Co*. *dwaya* (CBS 261.77) with high support value (100% ML/1.00 PP, Figure 1). Thus, we identified this isolate as a new collection of *Co*. *dwaya* from a freshwater habitat.

**Figure 5:**
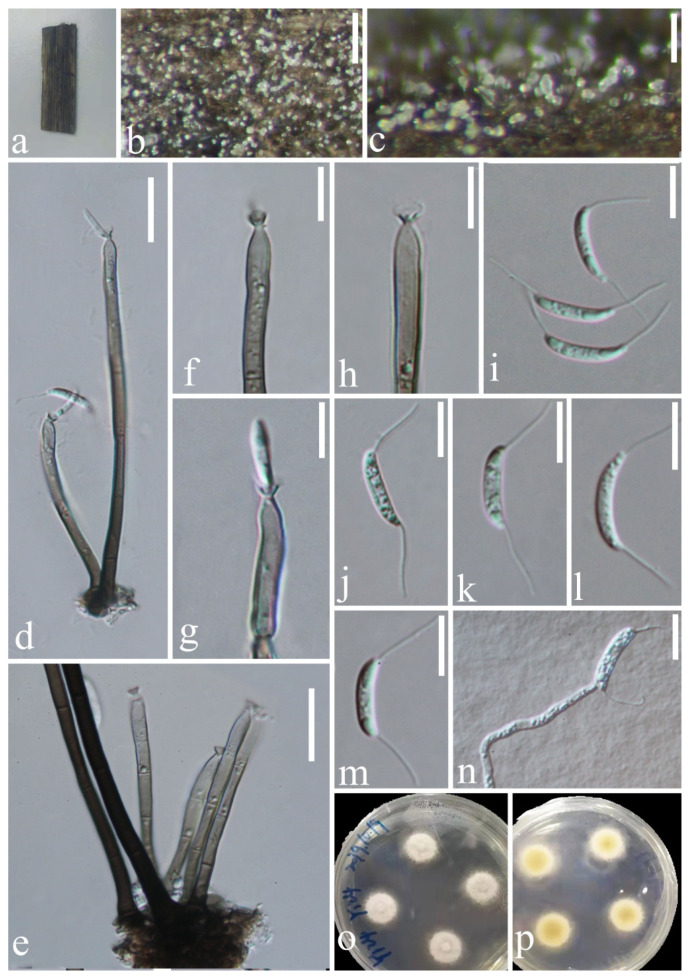
*Codinaea terminalis* (HKAS 123756). (**a**) Host material; (**b**,**c**) colonies on host surface; (**d**,**e**) conidiophores with conidiogenous cell; (**f**–**h**) conidiogenous cells; (**i**–**m**) conidia; (**n**) germinating condium; (**o**,**p**) culture on PDA from above and below. Scale bars: (**b**) 200 μm; (**c**) 100 μm; (**d**,**e**) 20 μm; (**f**–**o**) 10 μm.

*Codinaea terminalis* (C.G. Lin and K.D. Hyde) Réblová and Hern.-Restr., Journal of Fungi 7(12, no. 1097): 44 (2021). (Figure 5).

Index Fungorum number: IF 56706; Facesoffungi number: FoF 06287.

Holotype: MFLU 19-0214.

*Saprobic* on dead bamboo culms in freshwater habitats. Sexual morph: undetermined. Asexual morph: hyphomycetous. *Colonies* on natural substrate superficial, effuse, gregarious, white, shining, globose aggregated in a large mass. *Mycelium* mostly immersed, composed of septate, smooth, branched and pale brown hyphae. Setae 99–277 µm long, 3.1–4.7 µm wide at the base ($\bar{x}$ = 153 × 3.8 µm, *n* = 25), fertile, straight or slightly flexuous, dark brown at the base, paler towards the apex, septate, smooth-walled, unbranched, cylindrical. *Conidiophores* 34–62 × 3–4 µm ($\bar{x}$ = 48.3 × 3.4 µm, *n* = 20), macronematous, mononematous, erect, in groups from the mycelial knots from the bases of setae, cylindrical, slightly swollen at the base, straight or slightly flexuous, brown at the base becoming pale brown or subhyaline towards the apex, 2–4-septate, unbranched, smooth, guttules. *Conidiogenous cells* 14.5–36 µm long ($\bar{x}$ = 24 µm, *n* = 20), monophialidic, terminal, integrated, cylindrical, brown at the base, pale brown to subhyaline towards the apex, narrowing below the flared and conspicuous collarette. *Conidia* 13.5–16 × 2.5–3.3 µm ($\bar{x}$ = 14.5 × 2.9 µm, *n* = 35), aggregated in a glistening mass of conidia at the tip of conidiophores, amerospores, acrogenous, fusiform, mostly curved, guttules, rough-walled, hyaline, with hair-like and 9.6–11.5 µm-long appendages at both ends.

Culture characteristics: Conidia germinating on PDA within 15 h and germ tubes produced from conidia. Colonies growing on PDA, reaching 19 mm in diam. in 10 days at 25 °C, circular, flat with a protuberance in the center, white, entire margin with thin mycelia; pale yellow to white from center to edge in reverse and not producing pigmentation in culture.

Material examined: CHINA, Guizhou province, Zunyi city, Chishui county, Hushi town, Chishui Alsophila Natural Reserve (28°29′43″ N, 106°0′24″ E), on dead bamboo culms from a stream, 22 September 2019, Jing-Yi Zhang, Y124 (HKAS 123756 = GZAAS 22-0070); living culture, GZCC 22-0086. GenBank accession numbers: (LSU) ON502895, (ITS) ON502902.

Notes: *Codinaea terminalis* was introduced by Lin et al. [9], isolated from decaying leaves in China. The morphology of our isolate shares similar characters with the ex-type strain *Codinaea terminalis* (GZCC 18–0085). However, there are some differences in the size of the microstructure such as obviously longer setulae (9.6–11.5 µm vs. 4–9.5 μm). Our new isolate clustered among two *Dictyochaeta terminalis* strains (GZCC 18–0085 and GZCC19-0525) with strong statistical support (99% ML/1.00 PP, Figure 1). Thus, we identified this isolate as a new host record of *Co*. *terminalis* on dead bamboo culms in China.

**Figure 6:**
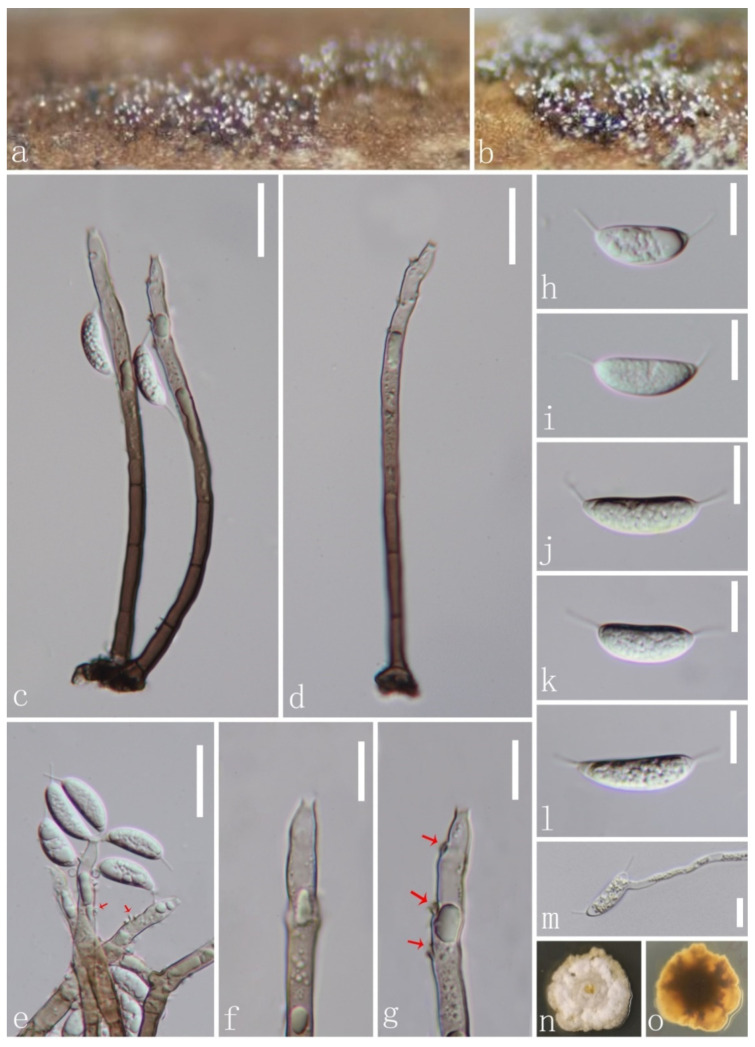
*Codinaeella hyalina* (HKAS 123757, holotype). (**a**,**b**) Colonies on woody substrate; (**c**,**d**) conidiophores and conidiogenous cells; (**e**–**g**) conidiogenous cells (arrows indicate collarettes); (**h**–**l**) conidia; (**m**) germinating conidium; (**n**,**o**) culture on PDA from above and below. Scale bars: (**c**–**e**) 20 μm; (**f**–**m**) 10 μm.

*Codinaeella hyalina* J.Y. Zhang and Y.Z. Lu, sp. nov. (Figure 6).

Index Fungorum number: IF559701; Facesoffungi number: FoF 11039.

Etymology: referring to its hyaline conidia.

Holotype: HKAS 123757.

*Saprobic* on decaying wood submerged in freshwater habitats. Sexual morph: undetermined. Asexual morph: hyphomycetous. *Colonies* on natural substrate superficial, effuse, mostly single, or arise in groups of 2–3 from knots of hyphal cells, dark brown, with glistening mass of conidia at the apex of conidiophores. *Mycelium* mostly immersed, composed of pale brown, septate hyphae. *Conidiophores* 93–143 × 3.4–5.1 μm ($\bar{x}$ = 121 × 4.4 μm), macronematous, mononematous, single or in small groups, erect, straight or slightly flexuous, smooth, dark brown at the base, becoming paler to subhyaline towards the apex, 2–7-septate, smooth, guttulate. *Conidiogenous cells* 23.6–68 × 3.2–5.5 μm ($\bar{x}$ = 50.3 × 4.3 μm), mono- or polyphialidic, with discrete, lateral phialides, integrated, terminal, with lateral openings formed by successive sympodial elongation, cylindrical to cylindrical–lageniform, with funnel-shaped collarettes, brown at the base and becoming subhyaline to hyaline towards the apex, smooth-walled. *Conidia* 15.9–21.6 × 5.8–7 μm ($\bar{x}$ = 18.6 × 6.3 μm, *n* = 20), amerospores, aseptate, often unilateral ventricose, reniform, aggregated in large and slimy mass, rough-walled, hyaline, with hair-like and 4–7 μm-long appendages at both ends.

Culture characteristics: Conidia germinating on PDA within 12 h. and germ tubes produced from conidia. Colonies growing on PDA, slow growth, reaching 20 mm in diam. in 20 days at 25 °C, circular, flat, with dense, white mycelium on the surface with undulate margin, from below olivaceous brown at the center, yellow at the edge and does not produce pigmentation in culture.

Material examined: CHINA, Guizhou province, Zunyi city, Xishui county, Taolin town, Tianlong, on submerged decaying wood in a freshwater stream, 13 February 2021, Jian Ma, TL24 (HKAS 123757, holotype; GZAAS22-0073 isotype), ex-type living culture, GZCC 22-0082. GenBank accession numbers: (LSU) ON502891, (ITS) ON502898.

Notes: Following BLASTn searches, the closest matches of our new isolate *Codinaeella hyalina* of ITS and LSU sequence data were *Codinaeella minuta* 417E (MZ078594, 96.57% similarity) and *Cod*. *minuta* ATCC 20960 (OL654146, 99.08% similarity), respectively. In the phylogeny (Figure 1), our new isolate formed a separate clade, and is basal to other species of *Codinaeella* with a high bootstrap support value (100% ML/1.00 PP). Morphologically, our new isolate shares similar characters to *Cod*. *mimusopis* in having macronematous, mononematous conidiophores, conidiogenous cell with lateral phialides and hyaline, aseptate conidia with filiform appendages at both ends [16,30]. However, our new species *Cod*. *hyalina* differs from *Cod*. *mimusopis* by having wider conidiogenous cells (3.2–5.5 μm vs. 3(–3.5) μm) and wider reniform conidia (15.9–21.6 × 5.8–7 μm vs. 16–18(–20) × 2.5–3(–3.5) μm). Additionally, *Cod*. *hyalina* differs from existing species of *Codinaeella* in having obviously wider conidia. Therefore, we introduce *Cod*. *hyalina* as a new species based on its distinct morphology and phylogenetic evidence.

**Figure 7:**
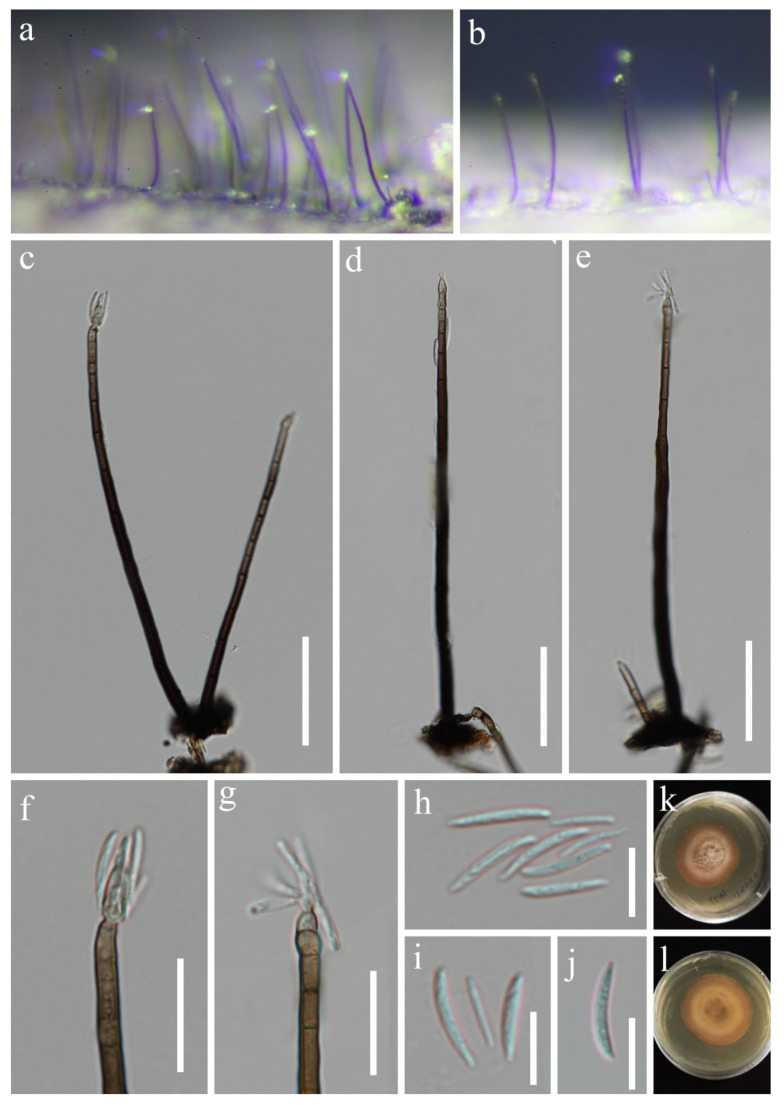
*Dictyochaeta guizhouensis* (HKAS 123753, holotype). (**a**,**b**) Colonies on dead wood; (**c**–**e**) conidiophores; (**f**,**g**) conidiogenous cells; (**h**–**j**) conidia; (**k**,**l**) culture on PDA from above and below. Scale bars: (**c**–**e**) 50 μm; (**f**,**g**) 20 μm; (**h**–**j**) 10 μm.

*Dictyochaeta guizhouensis* J.Y. Zhang and Y.Z. Lu, sp. nov. (Figure 7).

Index Fungorum number: IF559699; Facesoffungi number: FoF 11040.

Etymology: referring to the province “Guizhou”, where this species was collected.

Holotype: HKAS 123753.

*Saprobic* on dead wood in land. Sexual morph: undetermined. Asexual morph: hyphomycetous. *Colonies* on natural substrate superficial, effuse, gregarious, hairy, brown. *Conidiophores* 133–240 × 4–6.8 µm ($\bar{x}$ = 170.7 × 4.8 µm, *n* = 10), macronematous, mononematous, erect, straight, unbranched, multiseptate, smooth, dark brown at the base, becoming pale brown towards the apex. *Conidiogenous cells* 8.6–20 × 2.9–4.8 µm ($\bar{x}$ = 14.8 × 3.8 µm, *n* = 10), polyphialidic, terminal, integrated, with flared collarette, cylindrical, pale brown to subhyaline or hyaline towards the apex. *Conidia* 13–16 × 1.5–2.2 µm ($\bar{x}$ = 15 × 1.8 µm, *n* = 20), aggregated in a glistening and small mass of conidia at the tip of conidiophores, amerospores, acrogenous, asymmetrical, falcate, straight or slightly curved, often rounded at the apex, tapering toward the basal end, rough-walled, hyaline.

Cultural characteristics: Conidia germinating on PDA within 15 h and germ tubes produced from conidia. Colonies grew on PDA medium at 25 °C, circular, with an entire margin, umbonate, with dense, white mycelium in the middle and yellowish-brown mycelium in the outer ring on the surface; in contrast, they are cream yellow in the middle, while yellowish-brown in the center and the outer ring.

Material examined: CHINA, Guizhou province, Xishui county, Xishui Reserve, 7 September 2020, Yong-Zhong Lu, XSBHq(011) (HKAS 123753, holotype; GZAAS 22-0074, isotype), ex-type living culture, GZCC 22-0083. GenBank accession numbers: (LSU) ON502892, (ITS) ON502899.

Notes: In a BLASTn search in GenBank, the closest match to the LSU and ITS sequences of our new isolate (GZCC 22-0083) exhibited 100% similarity across 100% of the query sequence and 98.49% similarity across 90% of the query sequence to *Dictyochaeta* sp. (strain CBS 138684), respectively. The phylogenetic tree depicted that our new collection is closely related to unidentified taxon *Dictyochaeta* sp. (CBS 138684) with 97% ML/1.00 PP support (Figure 1). *Dictyochaeta* sp. (CBS 138684) was described by Réblová et al. [14] with its sexual and asexual morphs. However, there was insufficient evidence of morphological characteristics due to a sample ageing; therefore, *Dictyochaeta* sp. (CBS 138684) was treated as an unidentified species [14]. Our new isolate formed an asexual morph in the natural substrate and is characterized by a lack of setae, microconidia and polyphialidic conidiohenous cells with conspicuous collarette, which can be differentiated from *Dictyochaeta* sp. (CBS 138684). Given the remarkable differences in morphology between our new collection (HKAS 123753) and *Dictyochaeta* sp. (CBS 138684), we maintain *Dictyochaeta* sp. as an unidentified species and introduce our new collection as a novel species in *Dictyochaeta*.

**Figure 8:**
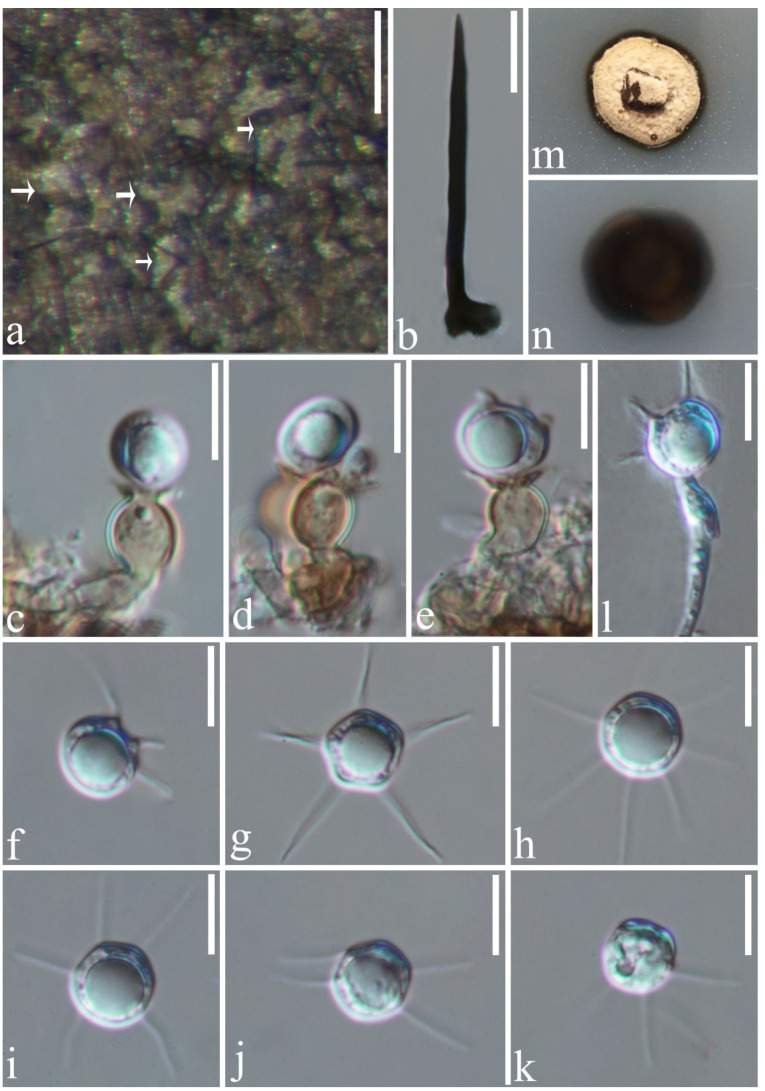
*Paragaeumannomyces guttulatus* (HKAS 123755, holotype). (**a**) Colonies (arrow) on host surface; (**b**) seta; (**c**–**e**) conidiogenous cell with conidia; (**f**–**k**) conidia; (**l**) germinating conidium; (**m**,**n**) colonies on PDA from above and below. Scale bars: (**a**) 100 μm; (**b**) 20 μm; (**c**–**l**) 10 μm.

*Paragaeumannomyces guttulatus* J.Y. Zhang and Y.Z. Lu, sp. nov. (Figure 8).

Index Fungorum number: IF559700; Facesoffungi number: FoF 11041.

Etymology: referring to its conidia with big guttules.

Holotype: HKAS 123755.

*Saprobic* on dead bamboo culms in freshwater habitats. Sexual morph: undetermined. Asexual morph: hyphomycetous. *Colonies* on natural substrate superficial, effuse, gregarious. *Mycelium* composed of partly immersed, partly superficial, septate, pale brown hyphae. *Setae* scattered over entire colonies, aseptate, dark brown, stiff, pointed, 65–110.5 μm long ($\bar{x}$ = 85 μm, *n* = 13). *Conidiophores* often reduced to conidiogenous cells, semi-macronematous to micronematous, mononematous, flexuous, unbranched, pale to moderately brown, thin-walled. *Conidiogenous cells* 6.4–10.3 × 5.4–8 μm ($\bar{x}$ = 8 × 6.5 µm, *n* = 20), monophialidic, flask-shaped, cup-shaped collarette, hyaline to light brown, guttulate. *Conidia* 9.4–11.5 μm diam. ($\bar{x}$ = 10.5 μm, *n* = 30), enteroblastic, acrogenous, aseptate, hyaline, globose to subglobose or ellipsoid, guttulate, with 2–6 hair-like and 8.8–13.5 µm-long appendages.

Culture characteristics: conidia germinating on PDA within 15 h and the hyaline germ tubes germinates from a point of the conidia. Colonies on MEA at 26 °C, slow growth, reach 5 mm in diam. in 30 days, circular, flat, with entire margin, taupe brown from above, while dark brown in reverse, and do not produce pigmentation in culture.

Material examined: CHINA, Guizhou province, Zunyi city, Chishui county, Hushi town, Chishui Alsophila Natural Reserve (28°29′43″ N, 106°0′24″ E), on dead bamboo culms from a freshwater stream, 22 September 2019, Jing-Yi Zhang, Y132 (HKAS 123755, holotype; GZAAS 22-0076, isotype); ex-type living culture, GZCC 22-0084. GenBank accession numbers: (LSU) ON502893, (ITS) ON502900.

Notes: Following BLASTn searches, the closest match to our new collection *Paragaeumannomyces guttulatus* is *Chaetosphaeria panamensis* (LSU, MT118218, 98.46% shared identity; ITS, KY212752, 92.68%). The phylogenetic results confirm that our new isolate *P*. *guttulatus* formed a separate clade within *Paragaeumannomyces* and shared a sister relationship with *P*. *panamensis* (S.M.H. 3596) with 94% ML/1.00 PP support (Figure 1). *Chaetosphaeria panamensis* was introduced by Huhndorf and Fernández [4] with sexual and asexual morphs. Later, *Chaetosphaeria panamensis* was transferred to the *Paragaeumannomyces* (*P*. *panamensis*) by Réblová et al. [29] based on generic concept and phylogenetic analysis. Morphologically, our new species *P*. *guttulatus* formed an asexual morph in the natural substrate and is similar to *P*. *panamensis* in having a “*Craspedodidymum*”-like asexual morph. However, *Paragaeumannomyces guttulatus* differs from *P*. *panamensis* in having bigger conidiogenous cells and smaller and rounder conidia with longer appendages [4,6]. Thus, we introduce *P*. *guttulatus* as a new species based on morphological and phylogenetic analyses.

**Figure 9:**
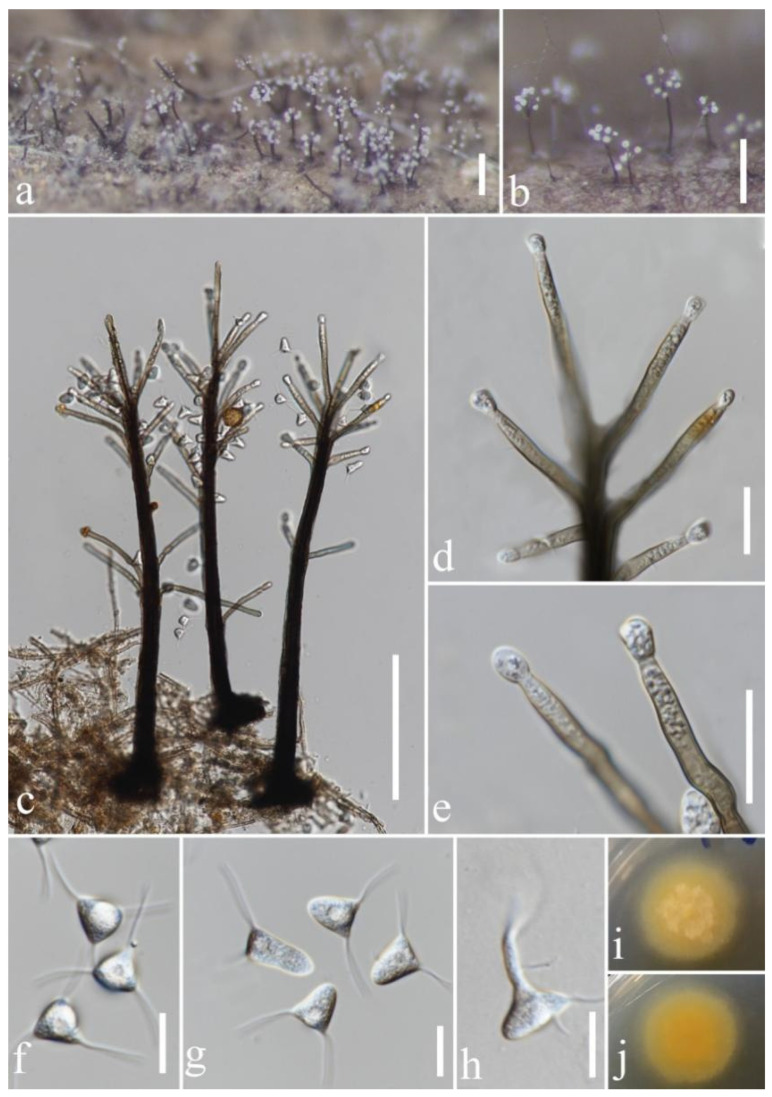
*Phialosporostilbe scutiformis* (MFLU 22-0077). (**a**,**b**) Colonies on woody substrate; (**c**) conidiophores; (**d**,**e**) conidiogenous cells with conidia; (**f**,**g**) conidia; (**h**) germinated conidium; (**i**,**j**) culture on PDA from above and below. Scale bars: (**a**,**b**) 200 μm; (**c**) 100 μm; (**d**,**e**) 20 μm; (**f**–**h**) 10 μm.

*Phialosporostilbe scutiformis* N.G. Liu, J. Yang and K.D. Hyde (2019). (Figure 9).

Index Fungorum number: IF555325; Facesoffungi number: FoF 04869.

Holotype: MFLU 18-1502.

*Saprobic* on decaying wood submerged in freshwater habitats. Sexual morph: undetermined. Asexual morph: hyphomycetous. *Colonies* on natural substrate superficial, effuse, scattered or gregarious, white, with glistening mass of conidia surround the conidiophores. *Mycelium* 2.1–3.2 μm ($\bar{x}$ = 2.7 μm) wide, composed of partly immersed, partly superficial, septate, brown hyphae. *Setae* 247–358 μm ($\bar{x}$ = 309 μm), rarely fertilizable, straight or slightly flexuous, dark brown at the base, paler towards the apex, mutiseptate, smooth-walled, unbranched, cylindrical; the lower part is surrounded and compacted by conidiophores. *Conidiophores* macronematous, synnematous, 11–13.7 μm ($\bar{x}$ = 12.5 μm) wide at the synnemata, always spread laterally along the upper half of the synnemata, erect, flexuous, unbranched, septate, smooth-walled, dark brown at the base, becoming pale brown and slightly tapering towards the apex, cylindrical. *Conidiogenous cells* 26–54 × 3.4–5.1 µm ($\bar{x}$ = 36 × 4.4 μm), monophialidic, terminal, integrated, clavate, thick-walled, rough, brown to subhyaline towards the apex, with an inconspicuous apical collarette. *Conidia* 6–7.9 μm ($\bar{x}$ = 7.1 μm, *n* = 20) long at each above edge, 9–14.4 μm ($\bar{x}$ = 10.8 μm, *n* = 20) long at the side, amerospores, acrogenous, round–tetrahedral, triangular from above, rough-walled, hyaline, with (6.5–)9.4–14 µm ($\bar{x}$ = 10.8 μm, *n* = 20)-long appendages at each corner.

Cultural characteristics: Conidia germinating on PDA within 15 h and germ tubes produced from the corner of conidia. Colonies on PDA medium, circular, reaching 13 mm in diam. in 10 days at 25 °C, entirely to slightly filamentous, with dense, yellowish-brown mycelium in the middle and thin, slightly yellow mycelium in the edge; yellowish in reverse and not producing pigmentation in culture.

Material examined: THAILAND, Chiang Rai province, on submerged decaying wood in a freshwater stream, 6 March 2021, Jing-Yi Zhang, Y269-2 (MFLU 22-0077); living culture, MFLUCC 22-0053.

GenBank accession numbers: (LSU) ON678145, (ITS) ON678180.

Notes: *Phialosporostilbe scutiformis* was introduced by Yang et al. [36] based on two specimens collected from freshwater habitats in China and Thailand. A BLASTn search of the ITS and LSU sequence data indicated that our new isolate is closely related to *P. scutiformis* (MFLUCC 17-0227) with 100% similarity. The morphology of our isolate and the paratype specimen of *P*. *scutiformis* (HKAS 102205) are indistinguishable, except that the big guttule in conidia was not observed in our collection. This may be due to the different observation periods. Phylogenetic analysis confirmed that our isolate MFLUCC 22-0053 grouped with *Phialosporostilbe scutiformis* strains (MFLUCC 17-0227 and MFLUCC 18-1288) with strong bootstrap support (99% ML/1.00 PP, Figure 1). Therefore, we identified this isolate as a new collection of *P*. *scutiformis* based on identical morphology and phylogenetic analysis.

## 4. Discussion

Most Chaetosphaeriaceous species are saprobic on wood or decaying plants from terrestrial and freshwater habitats, sometimes occurring on soil, while some are fungicolous taxa [1,2,6,11,12,14,20,29,56,57,58]. *Chaetosphaeria mangrovei* was only one species reported from the marine habitat [20]. In this study, five new species among eight newly collections, namely *Chaetosphaeria obovoidea*, *Codinaea aseptata*, *Codinaeella hyalina*, *Dictyochaeta guizhouensis* and *Paragaeumannomyces guttulatus*, were documented from China. This increased the number of Chaetosphaeriaceous species and exhibited high diversity, and undiscovered in China. Additionally, three previously known species including a new host record and two new collections were described and identified based on morphological comparison and phylogenetic analysis of a combined LSU and ITS sequence dataset.

The tree topologies resulting from phylogenetic reconstruction revealed Chaetosphaeriaceae are a well-resolved family (Figure 1). However, phylogenetic relationships of some genera in Chaetosphaeriaceae are problematic and uncertain [6,9,13,14,15,16,36]. The type genus *Chaetosphaeria* (Chaetosphaeriaceae, Chaetosphaeriales) is speciose and phylogenetically unresolved [1,3,4,5,6,9,10,22,29]. Its sexual morph is characterized by black, papillate ascomata, persistent paraphyses, clavate to cylindrical and unitunicate asci with a shallow, refractive, J-apical ring and hyaline, allantoid or ellipsoid, fusiform to filiform curved, septate ascospores with guttules [6,11,17,19,21,57,59]. The asexual morph is described as hyphomycetes with diversity, and is considered to be able to distinguish *Chaetosphaeria* species [3,10,11,20,23,51,59]. However, the high variability of asexual morphology complicates the phylogenetic placement of *Chaetosphaeria* [9,10,20,51]. Therefore, further studies on a complicated teleomorph–anamorph relationship are warranted, alongside molecular support to confirm the phylogenetic status of *Chaetosphaeria*.

## Figures and Tables

**Table 1:** Taxa, isolate information and GenBank accession numbers for new collections determined for this study.

Taxon	Specimen	Status	Country	Host	Habitat	GenBank Accessions	
						ITS	LSU
*Chaetosphaeria obovoidea*	HKAS 123765	H	China	Decaying wood	F	ON502901	ON502894
*Codinaea aseptata*	HKAS 123758	H	China	Decaying wood	F	ON502897	ON502890
*Codinaea dwaya*	GZAAS 22-0071		China	Decaying wood	F	ON502896	ON502889
*Codinaea terminalis*	HKAS 123756		China	Dead bamboo culms	F	ON502902	ON502895
*Codinaeella hyalina*	HKAS 123757	H	China	Decaying wood	F	ON502898	ON502891
*Dictyochaeta guizhouensis*	HKAS 123753	H	China	Decaying wood	T	ON502899	ON502892
*Paragaeumannomyces guttulatus*	HKAS 123755	H	China	Dead bamboo culms	F	ON502900	ON502893
*Phialosporostilbe scutiformis*	MFLU 22-0077		Thailand	Decaying wood	F	ON678180	ON678145

Note: status: H denotes holotype; habitat: F denotes freshwater habitat and T denotes terrestrial habitat.

**Table 2:** Taxa used in this study and their GenBank accession numbers.

Taxon	Strain		GenBank Accessions	
		Status	ITS	LSU
*Achrochaeta talbotii*	ICMP 15161		MT454480	MT454495
*Adautomilanezia caesalpiniae*	CC-LAMIC 102/12	T	KX821777	KU170671
*Anacacumisporium appendiculatum*	HMAS 245593	T	KP347129	KT001553
*Arcuatospora nouae zelandiae*	CBS 109474	T	MW984569	MW984552
*Arcuatospora seorsa*	CBS 147510	T	MW984572	MW984555
*Brunneodinemasporium brasiliense*	CBS 112007	T	JQ889272	JQ889288
*Brunneodinemasporium jonesii*	GZCC 16-0050	T	KY026058	KY026055
*Calvolachnella guaviyunis*	CBS 134695	T	KJ834524	KJ834525
*Catenularia minor*	PRM 900544	T	MW987827	MW987822
*Catenularia cubensis*	SMH 3258		MW987826	N/A
*Chaetosphaeria acutata*	CBS 101312		AF178553	AF178553
*Chaetosphaeria biapiculata*	SMH 3074		N/A	AF466065
*Chaetosphaeria caesariata*	SMH 2794		N/A	AF466060
*Chaetosphaeria chalaroides*	SMH 2018		N/A	AY017372
*Chaetosphaeria chlorotunicata*	SMH 1565	T	N/A	AF466064
*Chaetosphaeria conirostris*	SMH 2183		N/A	AF466066
*Chaetosphaeria curvispora*	CBS 125555		MH863562	MH875040
*Chaetosphaeria cylindrospora*	SMH 3568	T	N/A	AY017373
*Chaetosphaeria decastyla*	SMH 2629		N/A	AF466068
*Chaetosphaeria dilabens*	CBS 712 88		AF178557	AF178557
*Chaetosphaeria fennica*	CBS 101641		AF178562	AF178562
*Chaetosphaeria fusiformis*	CBS 101429		AF178554	AF178554
*Chaetosphaeria hebetiseta*	CBS 102340	T	AF178549	AF178549
*Chaetosphaeria innumera*	M R 1175		AF178551	AF178551
*Chaetosphaeria jonesii*	MFLUCC 15-1015	T	NR_154841	KY212761
*Chaetosphaeria lateriphiala*	SMH 2629		N/A	AF466070
*Chaetosphaeria lentomita*	MR 1265		AF178548	AF178548
*Chaetosphaeria lignomollis*	SMH 3015	T	EU037896	AF466073
*Chaetosphaeria luquillensis*	SMH 2973		N/A	AF466074
*Chaetosphaeria mangrovei*	MCD 069	T	MG813821	MG813820
*Chaetosphaeria metallicans*	PDD 92539	T	NR_119668	NG_058757
*Chaetosphaeria minuta*	SMH 3396		N/A	AF466075
*Chaetosphaeria myriocarpa*	CBS 264.76		AF178552	AF178552
*Chaetosphaeria preussii*	CBS 262.76		AF178561	AF178561
*Chaetosphaeria pygmaea*	MR 1365		AF178545	AF178545
*Chaetosphaeria rivularia*	CBS 127686	T	KR347356	KR347357
*Chaetosphaeria* sp. JEH 2019	JAUCC 2961		MN619651	MN607223
*Chaetosphaeria spinosa*	SMH 2754		N/A	AF466079
*Chaetosphaeria sylvatica*	SMH 2893		N/A	AF279419
*Chaetosphaeria tropicalis*	SMH 1267		N/A	AF279418
*Chalarodes obpyramidata*	PDD 119364		MW987828	MW987823
*Chloridium caesium*	CBS 102339		AF178564	AF178564
*Chloridium gonytrichii*	CBS 195.60		MH857954	MH869503
*Chloridium virescens*	CBS 152.53		MH857142	MH868678
*Codinaea acaciae*	OTU5		KY965397	N/A
*Codinaea amazonensis*	MUCL 41171		OL654076	OL654133
*Codinaea assamica*	CBS 242.66		MH858788	MH870426
*Codinaea assamica*	CBS 139907	T	OL654077	OL654134
*Codinaea dwaya*	CBS 261.77	T	OL654078	OL654135
*Codinaea ellipsoidea*	MFLUCC 18-1574	T	MK828628	MK835828
*Codinaea fertilis*	CBS 242.66		OL654079	OL654136
*Codinaea gonytrichodes*	CBS 593.93		AF178556	AF178556
*Codinaea lignicola*	DLUCC 0899	T	MK828630	MK835830
*Codinaea pandanicola*	KUMCC 16-0153	T	MH388338	MH376710
*Codinaea paniculata*	CBS 145098	T	MT118230	MT118201
*Codinaea phasma*	CBS 147516	T	OL654081	OL654138
*Codinaea siamensis*	CBS 194.96		OL654082	OL654139
*Codinaea siamensis*	MFLUCC 15-0614	T	KX609955	KX609952
*Codinaea terminalis*	GZCC19-0525		MW133883	N/A
*Codinaea terminalis*	GZCC 18-0085	T	MN104613	MN104624
*Codinaeella filamentosa*	CBS 147265		OL654083	OL654140
*Codinaeella lambertiae*	CBS 143419	T	OL654084	OL654141
*Codinaeella lutea*	CBS 146618	T	OL654085	OL654142
*Codinaeella mimusopis*	CBS 143435	T	MH107888	MH107935
*Codinaeella minuta*	CBS 298.61	T	OL654090	OL654147
*Codinaeella minuta*	CBS 966.69		AF178559	AF178559
*Codinaeella minuta*	417E		MZ078594	N/A
*Codinaeella parvilobata*	CBS 144536	T	OL654100	OL654157
*Codinaeella pini*	CBS 138866	T	KP004465	KP004493
*Codinaeella yunnanensis*	MFLUCC 17-0468		NR_168795	NG_068630
*Conicomyces pseudotransvaalensis*	HHUF 29956	T	LC001710	LC001708
*Cryptophiale udagawae*	GZCC 18-0047		MN104608	MN104619
*Dendrophoma cytisporoides*	CBS 144107		MT118234	MT118205
*Dictyochaeta callimorpha*	ICMP 15130		MT454483	MT454498
*Dictyochaeta detriticola*	ICMP 14948	T	MT454486	MT454501
*Dictyochaeta fuegiana*	ICMP 15153	T	MT454487	EF063574
*Dictyochaeta montana*	CBS 145342	T	MT454488	MT454502
*Dictyochaeta querna*	CBS 146103		MT454490	MT454504
*Dictyochaeta sp*	CBS 138684		MT454493	MT454507
*Dictyochaeta stratosa*	CBS 138739		NR 172308	MT454505
*Dinemasporium americanum*	CBS 127127	T	JQ889274	JQ889290
*Dinemasporium pseudoindicum*	CBS 127402	T	JQ889277	JQ889293
*Ellisembia aurea*	CBS 144403	T	MH836375	MH836376
*Ellisembia folliculata*	CBS 147152		OL654105	OL654162
*Eucalyptostroma eucalypti*	CBS 142074	T	KY173408	KY173500
*Flectospora laminata*	CBS 112964		MW984576	MW984558
*Flectospora* sp.	ICMP 23840		MW984577	MW984559
*Infundibulomyces cupulatus*	BCC 11929	T	EF113976	EF113979
*Infundibulomyces oblongisporus*	BCC 13400	T	EF113977	EF113980
*Kionochaeta castaneae*	GZCC 18-0025	T	MN104610	MN104621
*Kionochaeta microspora*	GZCC 18-0036	T	MN104607	MN104618
*Leptosporella arengae*	MFLUCC 15-0330	T	MG272255	MG272246
*Leptosporella bambusae*	MFLUCC 12-0846	T	KU940134	KU863122
*Menispora ciliata*	CBS 122131	T	EU488736	OL654165
*Menispora tortuosa*	CBS 117552		OL654110	OL654168
*Menisporopsis dushanensis*	GZCC 18-0084	T	MN104615	MN104626
*Menisporopsis theobromae*	MFLUCC 15 0055		KX609957	KX609954
*Multiguttulispora dimorpha*	CBS 140002		MW984582	MW984564
*Multiguttulispora triseptata*	IMI 353690		MW984584	MW984566
*Nawawia filiformis*	MFLUCC 17-2394		MH758196	MH758209
*Neopseudolachnella acutispora*	MAFF 244358	T	AB934065	AB934041
*Neopseudolachnella magnispora*	MAFF 244359	T	AB934066	AB934042
*Nimesporella capillacea*	IMI 358908	T	OL654114	OL654171
*Paliphora intermedia*	CBS 896 97		MH862682	EF204501
*Paragaeumannomyces abietinus*	CBS 145351	T	MT118235	MT118206
*Paragaeumannomyces albidus*	PDD 92537	T	EU037890	EU037898
*Paragaeumannomyces bombycinus*	PDD 92538	T	EU037892	N/A
*Paragaeumannomyces elegans*	PDD 118740	T	MT876580	N/A
*Paragaeumannomyces garethjonesii*	MFLUCC 15-1012	T	KY212751	KY212759
*Paragaeumannomyces granulatus*	ICMP 15133	T	MT876575	MT876577
*Paragaeumannomyces lapazianus*	SMH 2182		AY906945	MT118207
*Paragaeumannomyces longisporus*	ANM 1269		MT118239	MT118210
*Paragaeumannomyces panamensis*	SMH 3596	T	AY906948	MT118218
*Paragaeumannomyces raciborskii*	SMH 3119		AY906953	AY436402
*Paragaeumannomyces rubicundus*	SMH 3221	T	MT118242	MT118224
*Paragaeumannomyces sabinianus*	ILLS00121384	T	MT118243	MT118225
*Paragaeumannomyces smokiensis*	ILLS00121398	T	MT118240	MT118228
*Phialosporostilbe scutiformis*	MFLUCC 17-0227	T	MH758194	MH758207
*Phialosporostilbe scutiformis*	MFLUCC 18-1288		MH758199	MH758212
*Phialoturbella calva*	ICMP 23826	T	MW984585	MW984567
*Phialoturbella lunata*	MFLUCC 18-0642	T	NR 168796	MK835824
*Polynema podocarpi*	CBS 144415	T	MH327797	MH327833
*Pseudodinemasporium fabiforme*	CBS 140010	T	KR611889	KR611906
*Pseudolachnea fraxini*	CBS 113701	T	JQ889287	JQ889301
*Pseudolachnea hispidula*	MAFF 244365		AB934072	AB934048
*Pseudolachnella asymmetrica*	MAFF 244366		AB934073	AB934049
*Pseudolachnella scolecospora*	MAFF 244379		AB934086	AB934062
*Pyrigemmula aurantiaca*	CBS 126743	T	HM241692	HM241692
*Rattania setulifer*	GUFCC 15501		GU191794	HM171322
*Sporoschisma longicatenatum*	MFLUCC 16-0180	T	KX505871	KX358077
*Sporoschisma mirabile*	FMR 11247		HF677174	HF677183
*Stilbochaeta aquatica*	MFLUCC 15-0983	T	NR 158452	MH476569
*Stilbochaeta brevisetula*	ICMP 22549		OL654118	OL654175
*Stilbochaeta cangshanensis*	MFLUCC 17-2214	T	MK828632	MK835832
*Stilbochaeta malaysiana*	IMI 312436	T	OL654121	OL654178
*Striatosphaeria castanea*	CBS 145352	T	MT118244	MT118229
*Striatosphaeria codinaeophora*	SMH 1524		MT118245	AF466088
*Tainosphaeria jonesii*	GZCC 16-0065		KY026060	KY026057
*Tainosphaeria siamensis*	MFLUCC 15-0607	T	KX609956	KX609953
*Tainosphaeriella aquatica*	MFLUCC 17-2370		NR_173239	NG_079564
*Tainosphaeriella thailandensis*	MFLUCC 18-1282	T	MZ161198	MZ161196
*Thozetella nivea*	N/A		EU825201	EU825200
*Thozetella tocklaiensis*	CBS 378.58	T	MH857817	MH869349
*Xyladelphia longiseta*	SMH 1725		OL654131	AF279416
*Zanclospora iberica*	CBS 130426	T	KY853480	KY853544

Note: status: T denotes ex-type strains; “N/A” indicates no data are available in GenBank.

## Data Availability

All sequences generated in this study were submitted to GenBank.

## Supplementary Materials

The following supporting information can be downloaded at: https://www.mdpi.com/article/10.3390/jof8060643/s1. Table S1: taxa used in this study and their GenBank accession numbers.
